# Vancomycin Containing PDLLA and PLGA/β-TCP Inhibit Biofilm Formation but Do Not Stimulate Osteogenic Transformation of Human Mesenchymal Stem Cells

**DOI:** 10.3389/fsurg.2022.885241

**Published:** 2022-07-01

**Authors:** Berna Kankilic, Erdal Bayramli, Petek Korkusuz, Hakan Eroglu, Burcin Sener, Pelin Mutlu, Feza Korkusuz

**Affiliations:** ^1^ Graduate School of Natural and Applied Sciences, Middle East Technical University, Ankara, Turkey; ^2^ Department of Chemistry, Faculty of Arts and Sciences, Middle East Technical University, Ankara, Turkey; ^3^ Department of Histology and Embryology, Faculty of Medicine, Hacettepe University, Ankara, Turkey; ^4^ Department of Pharmaceutical Technology, Faculty of Pharmacy, Hacettepe University, Ankara, Turkey; ^5^ Department of Medical Microbiology, Faculty of Medicine, Hacettepe University, Ankara, Turkey; ^6^ Central Laboratory, Molecular Biology and Biotechnology R&D, Middle East Technical University, Ankara, Turkey; ^7^ Department of Sports Medicine, Faculty of Medicine, Hacettepe University, Ankara, Turkey

**Keywords:** vancomycin, PDLLA, PLGA, β-TCP, biofilm, bone signaling molecules

## Abstract

**Aims:**

Chronic osteomyelitis, including implant-related prosthetic joint infection, is extremely difficult to cure. We develop vancomycin containing release systems from poly(d,l-lactide) (PDLLA) and poly(d,l-lactide-co-glycolide) (PLGA) composites with beta-tricalcium phosphate (β-TCP) to treat methicillin-resistant *Staphylococcus aureus* osteomyelitis. We ask whether vancomycin containing PDLLA/β-TCP and PLGA/β-TCP composites will prevent early biofilm formation, allow cell proliferation and osteogenic differentiation, and stimulate osteogenic signaling molecules in the absence of an osteogenic medium.

**Methods:**

Composites were produced and characterized with scanning electron microscopy. *In vitro* vancomycin release was assessed for 6 weeks. Biofilm prevention was calculated by crystal violet staining. Human bone marrow-derived mesenchymal stem cells (hBM-MSCs) and osteosarcoma cell (SaOS-2) proliferation and differentiation were assessed with water soluble tetrazolium salt and alkaline phosphatase (ALP) staining. Real-time quantitative polymerase chain reaction defined osteogenic signaling molecules for hBM-MSCs.

**Results:**

Totally, 3.1 ± 0.2 mg and 3.4 ± 0.4 mg vancomycin released from PDLLA/β-TCP and the PLGA/β-TCP composites, respectively, and inhibited early biofilm formation. hBM-MSCs and SaOS-2 cells proliferated on the composites and stimulated ALP activity of cells. Runt-related transcription factor 2 (RUNX2) and SRY-Box transcription Factor 9 (SOX9) expressions were, however, lower with composites when compared with control.

**Conclusion:**

Vancomycin containing PDLLA/β-TCP and PLGA/β-TCP composites inhibited early biofilm formation and proliferated and differentiated hBM-MSCs and SaOS-2 cells, but osteogenesis-related RUNX2 and SOX9 transcription factors were not strongly expressed in the absence of an osteogenic medium for 14 days.

## Introduction

Chronic osteomyelitis is a bone infection leading to tissue damage and destruction with severe local and systemic morbidity (1) and mortality (2). The incidence of periprosthetic joint infection (PJI), which is a specific type of osteomyelitis, is mostly recognized by biofilm formation on an implant by methicillin-resistant *Staphylococcus aureus* (MRSA) that is between 0.3% and 3.0%, and mortality may increase up to 18% at revision (3). Average hospitalization costs in the US can be between 25.000 and 32.000 USD (4), which necessitates the development of new treatment strategies for the prevention and treatment of PJI.

Combining antibiotics with poly-methylmethacrylate (PMMA) is the standard treatment for PJI (5); however, PMMA has several drawbacks such as being a nonbiodegradable polymer and triggering the necessity for a second surgery for its removal. As PMMA shows an exothermic reaction during polymerization, only heat-stable antibiotics can be used with this polymer (6). Degradable composites are, therefore, used these days (7) to minimize the disadvantages of the non-degrading biomaterials. These composites should be active against the pathogens involved in the infection, release antibiotics at least 10 times higher than the minimum inhibitory concentration, should be biocompatible, and stimulate bone formation (8). Poly(d,l-lactide) (PDLLA) and poly(d,l-lactide-co-glycolide) (PLGA) are biodegradable and biocompatible polymers generally used as carriers in drug delivery systems (9). The disadvantages of these polymers are their acidic products after the biodegradation. The acidic products decrease the pH of the environment and fasten further degradation. Also, these polymers have low cell adhesion potential (10). On the other hand, beta-tricalcium phosphate (β-TCP) is a biodegradable bioceramic used in local drug delivery systems due to its high solubility rate and faster degradation time (11, 12). It also shows osteointegration and osteoconduction properties (13).We previously studied (14, 15) vancomycin containing PDLLA/β-TCP on human bone marrow-derived mesenchymal stem cells (hBM-MSCs) and osteosarcoma cell (SaOS-2) *in vitro* and on rats with experimental implant-related osteomyelitis *in vivo* for its drug release capability and biocompatibility; however, we did not assess its osteogenic potential. PLGA (16, 17) was evaluated for its vancomycin release and delivery capacity against infection. PLGA was also assessed for its osteogenic potential in a study by Yoon et al. (18). β-TCP was studied as a drug carrier (19), and a study (20) focused on the osteogenic potential of the material previously. We hypothesized that vancomycin containing PDLLA/β-TCP and PLGA/β-TCP composites will stimulate osteogenesis due to its high β-TCP content. Our research questions were whether vancomycin containing PDLLA/β-TCP and PLGA/β-TCP composites may prevent early biofilm formation, allow cell proliferation and osteogenic mineralization, and stimulate osteogenic signaling molecule expression of hBM-MSCs in the absence of the osteogenic medium.

We aimed for the evaluation of vancomycin release from PDLLA/β-TCP and PLGA/β-TCP composites to prevent early MRSA biofilm inhibition. Cytocompatibility and mineralization capacity of these composites were further assessed by water soluble tetrazolium salt (WST) and alkaline phosphatase (ALP) staining. Osteogenic signaling molecule expression of hBM-MSCs cultured with composites were evaluated using real-time quantitative polymerase chain reaction (qRT-PCR).

## Materials and Methods

### Design

**Figure 1:**
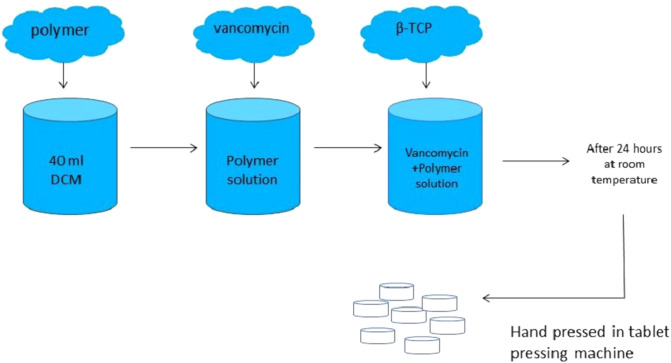
The design of the composites.

A controlled *in vitro* study was designed. Independent variables were groups and time, while dependent variables were vancomycin release, antibiotic susceptibility, early biofilm inhibition, cell proliferation, ALP activity, and osteogenic potential of the composites with qRT-PCR. The design of the composites is given in Figure 1.

### Materials

PLGA and PDLLA were purchased from Evonik Industries (Essen, Germany) and vancomycin hydrochloride was purchased from Zhejiang Medicine Co. Ltd. (Zhejiang, China). β-TCP was purchased from BMT Calsis (Ankara, Turkey). Dichloromethane (JT Baker, PA, USA) was used to dissolve the polymers. In order to prepare vancomycin containing PLGA/β-TCP composites, a total of 8,574 mg of PLGA was dissolved in 40 ml of dichloromethane. Then, 5,355 mg of vancomycin hydrochloride powder was added into the solution, followed by the addition of 16,071 mg β-TCP. The mixture was stirred on a magnetic stirrer with a closed lid and dried at room temperature for 24 h. After the evaporation of dichloromethane, the remaining powdery structure was ground on a porcelain mortar. For vancomycin containing PDLLA/β-TCP composites, all procedures mentioned above were repeated, but this time, 8,574 mg of PDLLA was used instead of PLGA. The powders were hand-pressed in a tablet-pressing machine, and totally, 149 vancomycin containing PLGA/β-TCP composite discs and 160 vancomycin containing PDLLA/β-TCP composite discs were obtained. The composite discs had a 3 mm height with 6 mm diameter. The final content ratios of the composites were 53.6% β-TCP, 28.6% polymer, and 17.8% vancomycin hydrochloride.

### Characterization of Composites

Surface topography and composition of the composites were characterized by using a scanning electron microscope (SEM; Nova Nanosem 430, Fei, OR, USA) with a built-in X-ray energy-dispersive spectrometer (EDS). The composites were fixed on supports and coated with gold film to obtain a conducting surface before the analysis.

The vancomycin containing composite discs were further evaluated by using a Fourier transform infrared microscope, which attenuated total reflection (FTIR-ATR; Bruker Alpha, Bruker, MA, USA). PLGA, PDLLA, β-TCP, and vancomycin were also analyzed to determine the similarities and differences between the composite discs and plain materials. The infrared spectrum was collected in the range of 4,000–400 cm^−1^ with a resolution of 4 cm^−1^ and a scan number of 24.

### *In Vitro* Vancomycin Release

Vancomycin containing composite discs from each group (*n* = 6) were immersed into a 50 ml phosphate buffered saline (PBS) containing polystyrene tubes. PBS was prepared by dissolving one PBS tablet (Oxoid, Hampshire, UK) in 100 ml distilled water. The tubes were placed into a hot water bath at 37°C and shaken constantly at 30 rpm. At predetermined time points (1, 2, 4, 8, 12, 24, 48, and 120 h and 1, 2, 3, 4, 5, and 6 weeks), 1 ml PBS was withdrawn and replaced with an equal amount of fresh PBS. The withdrawn solutions were analyzed spectrophotometrically with a Nanodrop ND 1000 spectrophotometer (Thermo Scientific, MA, USA) at 280 nm with a 1:10 dilution factor. A calibration curve for vancomycin was generated to calculate the amount of released vancomycin in the solution.

### Early Biofilm Inhibition Study

MRSA is the most common pathogen isolated from the infection site, but other pathogens like *Staphylococcus epidermidis*, coagulase-negative staphylococci, *Enterobacter* species, *Pseudomonas aeruginosa*, and *Mycobacterium* species are also responsible for osteomyelitis. *Staphylococcus aureus* is a gram positive, facultative anaerobe. It has a spherical shape with a 0.5–1.5 μm diameter and forms bead-like clusters when colonized. *S. aureus* is naturally found in human skin and nostrils. It attaches to the surface with its adhesins and exotoxins and many of its strains are capable of forming biofilm (21). Early biofilm inhibition capabilities of vancomycin containing composites discs were evaluated with the tissue culture plate method. A vancomycin containing composite disc from each group (*n* = 3) was put into a polystyrene tube containing 10 ml of PBS and placed in a hot water bath at 37°C and shaken constantly at 30 rpm. Every week, 1 ml PBS was withdrawn and replaced with an equal amount of fresh PBS until week 6. Each time point was carried out in triplicate. A slime-forming MRSA strain obtained from Hacettepe University, Faculty of Medicine, Department of Microbiology, was used for this study. Bacterial suspensions were pipetted into sterile glass tubes containing 2 ml of trypticase soy broth (Becton Dickinson, NJ, USA), and the bacterial cultures were set to 0.5 McFarland standard (1 × 10^−8 ^cfu/ml) with a benchtop turbidity meter (Grant Instruments, Cambridge, UK). A 200 μl bacterial suspension was inoculated to fresh 2 ml trypticase soy broth and incubated at 37°C overnight. On another day, bacteria suspension turbidity was checked with a benchtop turbidity meter and the bacteria suspension with 11.0 turbidity was poured into a fresh 48 ml trypticase soy broth. This process was done in duplicate. A 200 μl bacterial culture was added into each well of round bottom 96 well plates (Corning Costar, NY, USA), and 20 μl of drug release media collected from release studies were added to the wells. The plates were incubated at 37°C for 48 h. Trypticase soy broth without any bacteria was used as negative control, while bacterial culture without any release medium was used as positive control.

After incubation, tissue culture plates were turned upside down and the planktonic bacteria were poured out. The plates were washed three times with tap water (200 μl water for each well). After washing, 125 μl 0.1% crystal violet stain was added to each well, and the plates were incubated at room temperature for 10 min. The plates were shaken and the excess stain was poured out; again, the wells were washed twice with water. The plates were placed onto a paper towel and allowed to dry. Each well was filled with 200 μl 95% ethanol, and the plates were incubated at room temperature with closed lids for 15 min. The wells were gently mixed with pipetting, and a 125 μl ethanol-crystal violet mix from each well was placed into a new 96 well plate. The new plates were spectrophotometrically analyzed in ELISA reader (Tecan Sunrise, Mannedorf, Switzerland) at 620 nm.

### *In Vitro* Cell Culture Studies

Composites were evaluated for their proliferation and osteogenic potential in cell culture with hBM-MSCs (passage 6, Lonza, Basel, Switzerland) and SaOS-2 (passage 17, Sigma-Aldrich, MO, USA) cells at days 1, 3, and 7 in triplicate. hBM-MSCs and SaOS-2 cells without any composites were used as control groups. The cells were cultured with the hBM-MSCs or SaOS-2 medium according to cell type. The hBM-MSCs culture medium consisted of 52.8% Dulbecco's Modified Eagle Medium (DMEM) with 1 g/l glucose (Lonza, Basel, Switzerland), 35.2% MCDB-201 medium (Sigma-Aldrich, MO, USA), 10% heat-inactivated fetal bovine serum (FBS; Sigma-Aldrich, MO, USA), 1% penicillin/streptomycin solution (Biochrom AG, Berlin, Germany), and 1% l-glutamine (Biochrom AG, Berlin, Germany), while the SaOS-2 culture medium consisted of 89% DMEM with 4.5 g/l glucose (Sigma-Aldrich, MO, USA), 10% heat-inactivated FBS, and 1% penicillin/streptomycin solution. In every 3–4 days, the media were changed. The assay was done in 24-well cell culture plates (Corning Costar, NY, USA), with analysis for three different time points (on days 1, 3, and 7) in triplicate. In a 24-well cell culture plate, 12 wells were used for MSC, while the other 12 wells were used for SaOS-2 cells. A total of 7,500 cells were seeded on each well and then the composites were placed. The plates were incubated at 37°C with relative humidity under an atmosphere of 5% CO_2_. At predetermined time points, the medium was aspirated and a 500 μl fresh medium was added with 50 μl of cell proliferation agent WST-1 (Roche, Basel, Switzerland) for each well. The plates were incubated at 37°C with relative humidity under an atmosphere of 5% CO_2_ for 4 h. After incubation, a 110 μl 1:10 (v/v) WST-1 containing culture medium was pipetted into a flat bottom 96-well plate, and the absorbance of the wells was measured in ELISA reader (Tecan Sunrise, Mannedorf, Switzerland) at 450 nm with 620 nm reference wavelength. Early mineralization potential of the vancomycin containing composites was evaluated with ALP activity staining for hBM-MSCs and SaOS-2 cells. Cells were cultured with the hBM-MSCs or SaOS-2 medium according to the cell type, and on day 21, the medium was discarded and a 400 μl Alkaline Phosphatase Yellow Liquid substrate system for ELISA (Sigma-Aldrich, MO, USA) was added. The plate was incubated for 30 min, and 100 μl of 3 N sodium hydroxide (NaOH) was added to stop the reaction. A 200 μl final product was pipetted to a flat bottom 96-well plate and analyzed with an ELISA reader (Tecan Sunrise, Mannedorf, Switzerland) at 405 nm wavelength.

### qRT-PCR Assay

The hBM-MSCs (total 1.5 × 10^6^ cells) were cultured in T75 flasks (Corning Costar, NY, USA) with the hBM-MSCs medium at 37°C with relative humidity under an atmosphere of 5% CO_2_, and in every 3–4 days, the media were refreshed. When the cells reached 60%–70% confluency, culture media in three flasks were discarded and replaced with an osteogenic differentiation medium consisting of 10% FBS, 100 nM dexamethasone, 10 mM ß-glycerophosphate (Applichem, Germany), and 0.2 mM l-ascorbic acid (Sigma-Aldrich, MO, USA) in DMEM-LG. The remaining flasks were used for two different composite discs and extraction media were used for this purpose. Briefly, 33 composite discs from each group were incubated with 30 ml of the hBM-MSCs medium. After 14 days of incubation, the cells were trypsinized with 0.25% Trypsin-Ethylenediaminetetraacetic acid (EDTA) (Invitrogen, Gibco, UK) and suspended in 200 μl PBS. mRNA was isolated with a High Pure RNA Isolation Kit (Roche, Basel, Switzerland) and complementary DNA (cDNA) was synthesized with its kit (Roche, Basel, Switzerland). A 15 μl PCR mix and 5 μl cDNA were pipetted into each well of custom plate designed with different signaling molecules (Roche, Basel, Switzerland). The final PCR reaction was quantified in a Lightcycler 480 and its software was used to calculate the crossing point (*Cp*) for target and reference expression with the Advance Relative Quantification method. All target genes were normalized to housekeeping genes *ACTB* (beta actin), *GAPDH* (glyceraldehyde 3-phosphate dehydrogenase), and *G6PD* (glucose-6-phosphate dehydrogenase). The results were given as fold change corresponding to the hBM-MSCs control group according to ΔΔ*Ct* calculation. The sequences of primers are given in Table 1.

**Table 1:** Sequences of primers.

Gene name	Gene description	Forward primer sequence	Reverse primer sequence
*ALPL*	Alkaline phosphatase, liver/bone/kidney	AGAACCCCAAAGGCTTCTTC	CTTGGCTTTTCCTTCATGGT
*ANXA5*	Annexin A5	TCTTCGGAAGGCTATGAAAGG	GGGATGTCAACAGAGTCAGGA
*BGLAP*	Bone gamma-carboxyglutamate (gla) protein	CCAGCCCTATGGATGTGG	TTTTCAGATTCCTCTTCTGGAGTT
*BMP1*	Bone morphogenetic protein 1	TATGTGGAGGTCCGAGATGG	GAGTTTGGACCCGCAGAA
*BMP2*	Bone morphogenetic protein 2	GACTGCGGTCTCCTAAAGGTC	GGAAGCAGCAACGCTAGAAG
*BMP3*	Bone morphogenetic protein 3	CCCAAGTCCTTTGATGCCTA	TCTGGATGGTAGCATGATTTGA
*BMP4*	Bone morphogenetic protein 4	GAGGAAGGAAGATGCGAGAA	GCACTACGGAATGGCTCCT
*CDH11*	Cadherin 11, type 2,	CATCGTCATTCTCCTGGTCA	TCAAAGACAATGAGTGGTTCTTTC
*COL10A1*	Collagen, type X, alpha 1	CAGTTCTTCATTCCCTACACCA	AGGACTTCCGTAGCCTGGTT
*COL14A1*	Collagen, type XIV, alpha 1	GACCCCTCATCATGTTCTGC	ATGGCTTCCAGCTCATCTTG
*COL15A1*	Collagen, type XV, alpha 1	TGATGGTCGAGACATAATGACA	GGAGCCATGCCAAATGAC
*COL1A1*	Collagen, type I, alpha 1	AGGTGAAGCAGGCAAACCT	CTCGCCAGGGAAACCTCT
*COL1A2*	Collagen, type I, alpha 2	TCTGGAGAGGCTGGTACTGC	GAGCACCAAGAAGACCCTGA
*COL2A1*	Collagen, type II, alpha 1	TTTCAAGGCAATCCTGGTG	TCCAGGTTTTCCAGCTTCAC
*COL3A1*	Collagen, type III, alpha 1	ACTGGAGCACGGGGTCTT	TCCTGGTTTCCCACTTTCAC
*COL5A1*	Collagen, type V, alpha 1	TCTTGGCCCAAAGAAAACC	GGCGTCCACATAGGAGAGC
*COMP*	Cartilage oligomeric matrix protein	GGGTCCCCAATGAAAAGG	CCTTTTGGTCGTCGTTCTTC
*CTSK*	Cathepsin K	CGAAGCCAGACAACAGATTTC	AGAGCAAAGCTCACCACAGG
*EGF*	Epidermal growth factor	CCTCAGATGGGAAAACGTG	GTTCTTTAGATCAACTTCACCACCT
*EGFR*	Epidermal growth factor receptor	CAGCCACCCATATGTACCATC	AACTTTGGGCGACTATCTGC
*FGF1*	Fibroblast growth factor 1 (acidic)	AATCAGCCAAAGAGCCTGTC	CAAAACAGAGCAGGGAACTACC
*FGF2*	Fibroblast growth factor 2 (basic)	CCCGACGGCCGAGTTGAC	CACATTTAGAAGCCAGTAATCT
*FGFR1*	Fibroblast growth factor receptor 1	AAGATTGGCCCAGACAACC	GCACCTCCATCTCTTTGTCG
*FGFR2*	Fibroblast growth factor receptor 2	GACCCAAAATGGGAGTTTCC	GACCACTTGCCCAAAGCA
*IGF1*	Insulin-like growth factor 1	TGCTTTTGTGATTTCTTGAAGG	GCAGAGCTGGTGAAGGTGA
*IGF1R*	Insulin-like growth factor 1 receptor	TCAGCGCTGCTGATGTGT	GGCTCATGGTGATCTTCTCC
*IGF2*	Insulin-like growth factor 2	GCTGGCAGAGGAGTGTCC	GGGATTCCCATTGGTGTCT
*ITGB1*	Integrin, beta 1 (fibronectin receptor, antigen CD29 includes MDF2, MSK12)	CTTGGAACAGATCTGATGAATGA	TCCACAAATGAGCCAAATCC
*MMP2*	Matrix metallopeptidase 2	TATTTGATGGCATCGCTCAG	ACAGTCCGCCAAATGAACC
*MMP8*	Matrix metallopeptidase 8	GGGAACGCACTAACTTGACC	TTCAAAGGCATCCTTGATAGC
*PHEX*	Phosphate regulating endopeptidase homolog, X-linked	AGTGCATCCACCAACCAGAT	TTCCCCAAAAGAAAGGCTTC
*RUNX2*	Runt-related transcription factor 2	GCCTAGGCGCATTTCAGAT	CTGAGAGTGGAAGGCCAGAG
*SMAD1*	SMAD family member 1	TGTGTACTATACGTATGAGCTTTGTGA	TAACATCCTGGCGGTGGTA
*SMAD2*	SMAD family member 2	AAAGGGTGGGGAGCAGAATA	GAAGTTCAATCCAGCAAGGAGT
*SMAD3*	SMAD family member 3	GCATGAGCTTCGTCAAAGG	AATCCAGCAGGGGGTACTG
*SMAD4*	SMAD family member 4	TGGCCCAGGATCAGTAGGT	CATCAACACCAATTCCAGCA
*SOX9*	SRY (sex-determining region Y)-box 9	TACCCGCACTTGCACAAC	TCTCGCTCTCGTTCAGAAGTC
*TGFB1*	Transforming growth factor, beta 1	ACTACTACGCCAAGGAGGTCAC	TGCTTGAACTTGTCATAGATTTCG
*TGFB2*	Transforming growth factor, beta 2	GAAGAACTAGAAGCAAGATTTGCAG	TGATCACCACTGGTATATGTGGA
*TGFB3*	Transforming growth factor, beta 3	GCTTTGGACACCAATTACTGC	CCCAGATCCTGTCGGAAGT
*TGFBR1*	Transforming growth factor, beta receptor 1	AAATTGCTCGACGATGTTCC	CATAATAAGGCAGTTGGTAATCTTCA
*TGFBR2*	Transforming growth factor, beta receptor II	GACCAGAAATTCCCAGCTTCT	CAACGTCTCACACACCATCTG
*TWIST1*	Twist homolog 1 (Drosophila)	AGCTACGCCTTCTCGGTCT	TCCTTCTCTGGAAACAATGACA
*VDR*	Vitamin D (1,25-dihydroxyvitamin D3) receptor	CTTCTCTGGGGACTCCTCCT	TGGACGAGTCCATCATGTCT
*HPRT1*	Hypoxanthine phosphoribosyltransferase 1	TGACCTTGATTTATTTTGCATACC	CGAGCAAGACGTTCAGTCCT
*GDF10*	Growth differentiation factor 10	TGAATGGATAATCTCACCGAAA	GTTGGATGGACGAACGATCT
*ACTB*	Actin, beta	GGCCAGGTCATCACCATT	GGATGCCACAGGACTCCAT
*GAPDH*	Glyceraldehyde-3-phosphate dehydrogenase	CTCTGCTCCTCCTGTTCGAC	ACGACCAAATCCGTTGACTC
*G6PD*	Glucose-6-phosphate dehydrogenase	TCCATCAGTCGGATACACACA	CACCAGATGGTGGGGTAGAT
Control	Polymerase (RNA) II (DNA directed) polypeptide A, 220 kDa	CCTGAGTCCGGATGAACTG	GCCTCCCTCAGTCGTCTCT
Control	Polymerase (RNA) II (DNA directed)	GCAAATTCACCAAGAGAGACG	CACGTCGACAGGAACATCAG
Control	Polymerase (RNA) II (DNA directed) polypeptide A, 220kDa	TCCGTATTCGCATCATGAAC	TCATCCATCTTGTCCACCAC
Control	Transferrin receptor (p90, CD71)	TGGGTTTTTGTTACCTTTATGGTT	GGAGGTAACATGCAAATAATGTGA
Control	Transferrin receptor (p90, CD71)	TGGGTTTTTGTTACCTTTATGGTT	GGAGGTAACATGCAAATAATGTGA

### Statistical Analysis

All results were presented as average ± standard deviation and analyzed with SPSS 11.0. Statistically significant values were defined as *p* < 0.05 based on Student’s *t*-test. For determining the significance of the expression fold changes between the groups, the binary logarithm of the ΔΔ*Ct* values was calculated and ±two-fold changes were assigned as significant for the qRT-PCR study. The significant values are indicated in gray boxes in Table 2.

**Table 2:** The upregulation or downregulation of genes in the test groups according to control.

Fold up- or down-regulation according to control			
Gene	Osteogenic Medium + hBM-MSCs	PDLLA/β-TCP + hBM-MSCs	PLGA/β-TCP + hBM-MSCs
*ALPL*	3.44	−1.36	−0.37
*ANXA5*	0.17	−1.11	−0.73
*BGLAP*	1.33	−0.06	0.69
*BMP1*	2.11	−0.10	0.52
*BMP2*	1.78	2.18	3.36
*BMP3*	−1.29	−2.31	−1.12
*BMP4*	0.49	−1.16	−0.98
*CDH11*	−0.09	−0.88	−0.56
*COL10A1*	1.60	0.93	3.09
*COL14A1*	−2.06	−0.79	−0.70
*COL15A1*	−1.05	0.65	1.65
*COL1A1*	0.13	0.80	1.11
*COL1A2*	0.56	0.16	0.46
*COL3A1*	1.26	0.34	0.68
*COL5A1*	−0.89	−0.17	0.11
*COMP*	3.51	1.19	2.55
*CTSK*	2.41	0.49	1.14
*EGF*	−0.46	−1.37	−1.20
*EGFR*	−0.41	−1.23	−1.36
*FGF1*	−0.59	−0.57	−0.10
*FGF2*	−2.85	−0.74	−1.17
*FGFR1*	0.35	−0.03	0.19
*FGFR2*	−0.60	−0.70	−0.54
*IGF1*	0.76	1.93	2.53
*IGF1R*	0.13	−1.18	−1.19
*IGF2*	3.63	−1.72	−0.94
*ITGB1*	−0.11	−0.90	−0.63
*MMP2*	0.19	0.67	1.17
*MMP8*	5.72	1.12	1.82
*PHEX*	0.75	−2.91	−1.01
*RUNX2*	1.34	−0.60	−0.43
*SMAD1*	0.45	−2.26	−1.01
*SMAD2*	0.17	−0.87	−0.57
*SMAD3*	−1.26	−1.14	−1.66
*SMAD4*	0.30	−0.58	−0.44
*SOX9*	−1.38	0.01	−0.14
*TGFB1*	0.27	0.86	1.28
*TGFB2*	0.51	−1.74	−2.39
*TGFB3*	1.07	0.20	0.89
*TGFBR1*	−5.17	−4.85	−4.41
*TGFBR2*	1.30	−0.51	−0.26
*TWIST1*	0.85	0.35	−0.54
*VDR*	−0.59	2.59	2.02

*PDLLA, poly(d,l-lactide); PLGA, poly(d,l-lactide-co-glycolide); β-TCP, beta-tricalcium phosphate; hBM-MSCs, human bone marrow-derived mesenchymal stem cells.*

## Results

### Composite Characterization

**Figure 2:**
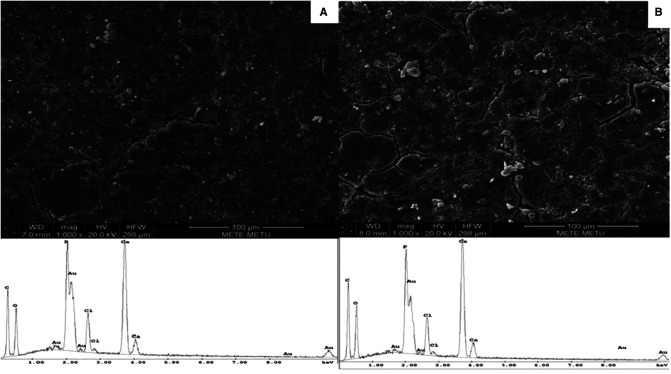
SEM micrographs (1,000× magnification) and EDS spectra of vancomycin containing (**A**) PDLLA/β-TCP and (**B**) PLGA/β-TCP composites. PDLLA, poly(d,l-lactide); PLGA, poly(d,l-lactide-co-glycolide); β-TCP, beta-tricalcium phosphate; EDS, energy-dispersive spectrometry; SEM, scanning electron microscope.

The surfaces of the composites contained micro cracks. The surface properties of the PDLLA/β-TCP and the PLGA/β-TCP composites were similar (Figure 2).

**Figure 3:**
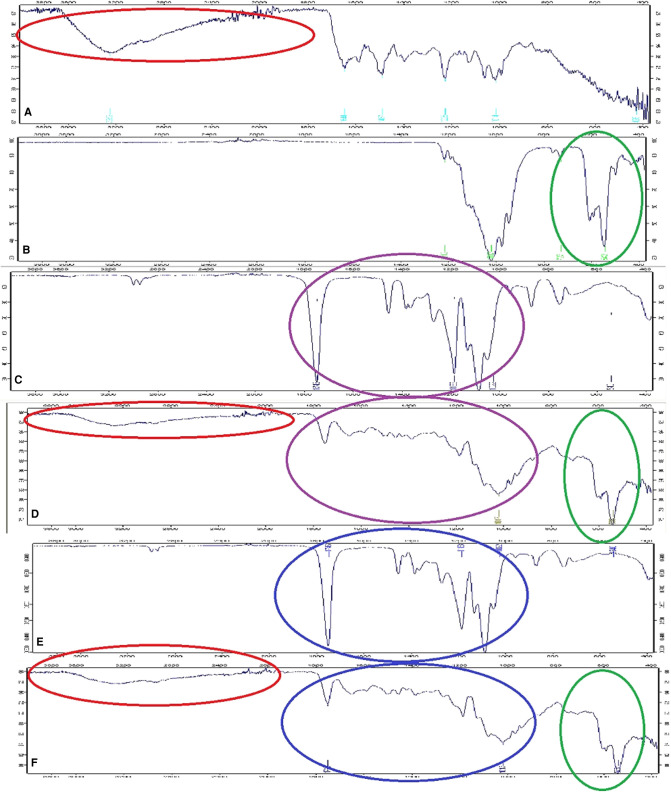
FTIR spectra of (**A**) vancomycin, (**B**) β-TCP, (**C**) PDLLA, (**D**) vancomycin containing PDLLA/β-TCP composite, (**E**) PLGA, and (**F**) vancomycin containing PLGA/β-TCP composite. PDLLA, poly(d,l-lactide); PLGA, poly(d,l-lactide-co-glycolide); β-TCP, beta-tricalcium phosphate.

The adsorption bands of vancomycin were recorded at 3,252, 1,644, 1,487, 1,225, 1,014, and 426 cm−^1^. The adsorption band at 3,252 cm^−1^ was for O–H stretching, while 1,644 cm^−1^ showed C=O stretching. The bands at 1,487 and 1,225 cm^−1^ pointed at C=C band and C–O–C band, respectively (22). The adsorption bands of β-TCP were found at 1,212 cm^−1^ (the pyrophosphate CPP group band), 1,017 cm^−1^ (C–O stretching), 727 cm^−1^ (P–O stretching), and 542 cm^−1^ (P–O bending) (23). The characteristic peaks of PDLLA and PLGA were found at 1,746 cm^−1^ (C=O band), 1,183 cm^−1^ (C–O band), 1,022 cm^−1^ (C–O band), and 540 cm^−1^ (C–H band) (24). The peaks were recorded at 1,749 cm^−1^ (C=O band), 1,017 cm^−1^ (C–O band), and 538 cm^−1^ in both vancomycin containing composites. The similarities of spectra were pointed in circles; the color red defined vancomycin, green defined β-TCP, purple defined PDLLA, and blue defined PLGA (Figure 3).

### Vancomycin Releasing Capacity of Composites

**Figure 4:**
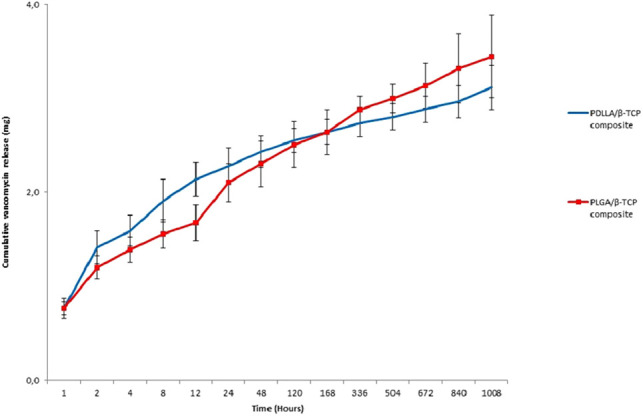
Cumulative released vancomycin amount from the composites in 6 weeks. PDLLA, poly(d,l-lactide); PLGA, poly(d,l-lactide-co-glycolide); β-TCP, beta-tricalcium phosphate.

Both PDLLA/β-TCP and PLGA/β-TCP composites maintained a sustained release of vancomycin for 6 weeks. The PDLLA/β-TCP composites released 2.3 ± 0.2 mg vancomycin, while the PLGA/β-TCP composites released 2.1 ± 0.2 mg in a day. After 6 weeks, cumulatively, 3.1 ± 0.2 and 3.4 ± 0.4 mg vancomycin were released from the PDLLA/β-TCP and the PLGA/β-TCP composites, respectively (Figure 4).

### Early Biofilm Inhibition Capacity

**Figure 5:**
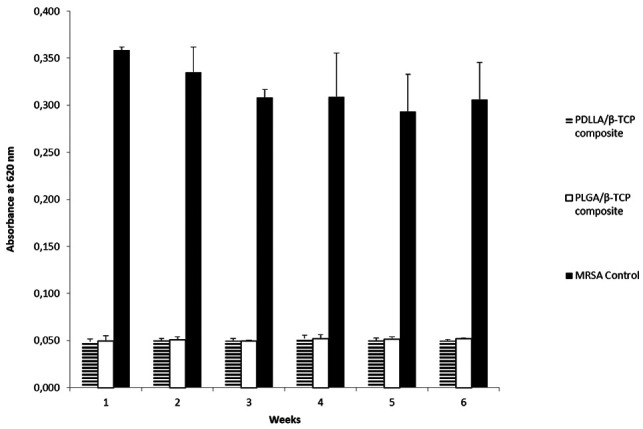
Absorbance results of bacterial control, PDLLA/β-TCP and PLGA/β-TCP composites at 620 nm (weeks are defining the time point of drug release media collected from release studies). PDLLA, poly(d,l-lactide); PLGA, poly(d,l-lactide-co-glycolide); β-TCP, beta-tricalcium phosphate; MRSA, methicillin-resistant *Staphylococcus aureus*.

In the biofilm inhibition study, there was a statistically significant difference between the composite groups and the bacterial control (*p* < 0.05). Released medium added to the bacterial suspensions inhibited early biofilm formation throughout 6 weeks (Figure 5).

### Cell Proliferation Capacity

**Figure 6:**
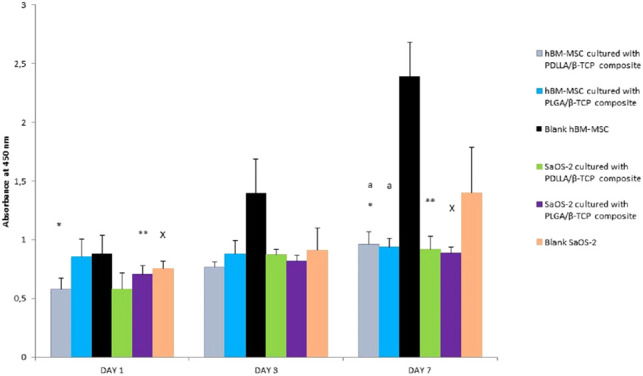
Proliferation of the MSC and SaOS-2 cells in days 1, 3, and 7 according to the absorbance of WST-1 at 450 nm. PDLLA, poly(d,l-lactide); PLGA, poly(d,l-lactide-co-glycolide); β-TCP, beta-tricalcium phosphate; SaOS-2, osteosarcoma cell; hBM-MSC, human bone marrow-derived mesenchymal stem cell; WST, water soluble tetrazolium salt.

Cells cultured on the composites proliferated, and there was a statistically significant difference for the PDLLA/β-TCP group between day 1 and day 7 for both cell lines (*p* = 0.01 for hBM-MSCs and *p* = 0.03 for SaOS-2, respectively). On the contrary, the PLGA/β-TCP group only showed a statistically significant difference between day 1 and day 7 for the SaOS-2 cell line (*p* = 0.03). Both composite groups showed a statistically significant difference versus blank hBM-MSCs on day 7 (*p* = 0.01), but there was no such significant difference for SaOS-2. There was no statistically significant difference between the groups for day 1 to day 3 or for day 3 to day 7 (Figure 6).

### Early Mineralization Potential of the Composites with ALP

**Figure 7:**
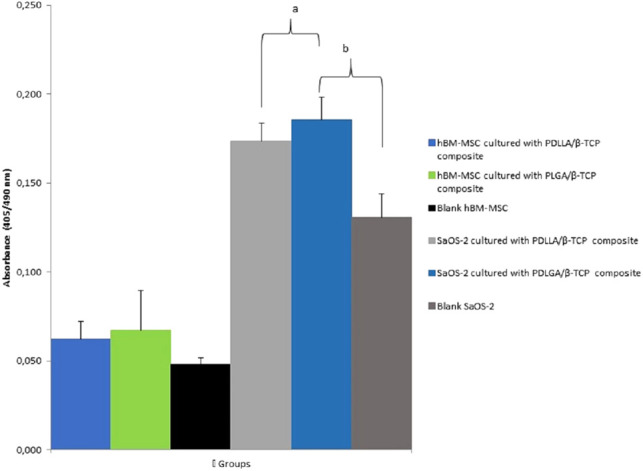
Alkaline phosphatase (ALP) activity of the groups at day 21 according to their absorbances at 405 nm. PDLLA, poly(d,l-lactide); PLGA, poly(d,l-lactide-co-glycolide); β-TCP, beta-tricalcium phosphate; SaOS-2, osteosarcoma cell; hBM-MSC, human bone marrow-derived mesenchymal stem cell.

PDLLA/β-TCP (*p* = 0.011) and the PLGA/β-TCP (*p* = 0.006) composites cultured with SaOS-2 cells presented a higher ALP activity compared with the SaOS-2 cell group without any composite. The ALP activity of the composites cultured with hBM-MSCs, however, was higher but was not statistically significant than the hBM-MSCs group (Figure 7).

### Osteogenic Capacity of the Cells Grown Together with Composites with qRT-PCR

The alkaline phosphatase (ALPL) upregulated 3.44-fold higher in the osteogenic medium group, while it downregulated in composite groups according to control. This downregulation, however, was statistically not significant. Bone morphogenetic protein-1 (BMP-1) upregulated significantly only in the osteogenic medium group, while bone morphogenetic protein-2 (BMP-2) was upregulated only in the composite groups. Bone morphogenetic protein-3 (BMP-3) downregulation was statistically significant only for PDLLA/β-TCP. Bone morphogenetic protein-4 (BMP-4) was not expressed significantly in any group. Collagen, type X, and alpha 1 (COL10A1) expressed significantly only in the PLGA/β-TCP group. The cartilage oligomeric matrix protein (COMP) was upregulated in each group, but its expression was significant only in the osteogenic medium and in the PLGA/β-TCP groups. Cathepsin K (CTSK), insulin-like growth factor 2 (IGF2), and matrix metallopeptidase 8 (MMP8) expressions upregulated only in the osteogenic medium group. The expression of insulin-like growth factor 1 (IGF1) increased in all groups, while it was significant for the PLGA/β-TCP group.

SMAD expressions decreased in the composite groups, which were significant only for SMAD1 in the PDLLA/β-TCP group.

Transforming growth factor, beta 2 (TGF-β2), downregulated in the PLGA/β-TCP group, while TGF-βR1 (receptor 1 of TGF-β) downregulated in all groups. Vitamin D receptor (VDR) downregulated in the osteogenic medium group; on the contrary, it was upregulated significantly in the composite groups. RUNX2 upregulated in the osteogenic medium group and downregulated in the composite groups; however, these expressions were not significant (Table 2).

## Discussion

MRSA chronic osteomyelitis is a devastating disease with limited cure, including long-term systemic antibiotic administration and repetitive surgeries (25). Poor blood circulation in the infection area and bone necrosis makes osteomyelitis a persistent disease, and treatment can hardly be achieved (26); so, local antibiotic delivery systems are generated (27). Various polymers, calcium-based composites, and manufacturing methods for local drug delivery systems reveal that an optimum system has not yet been produced (28–32). PDLLA, PLGA, and β-TCP are chosen to fabricate the composites since these materials are clinically used for a long time due to their safety and biocompatibility (33, 34). Booysen et al. searched for the cytotoxicity of vancomycin on hBM-MSCs and found that a high amount of vancomycin did not lead to any cytotoxicity as it did not inhibit the osteogenic differentiation (35).

Both PDLLA/β-TCP and PLGA/β-TCP composites released vancomycin for 6 weeks. The composites had smooth surfaces with microcracks. PDLLA/β-TCP composites released only 3.1 ± 0.2 mg of its vancomycin, while PLGA/β-TCP composites released 3.4 ± 0.4 mg. There was a slight difference between the released amounts, so the type of polymer used in this study did not have an impact on the release properties. On the contrary, the initial burst of vancomycin in 24 h was in line with a previous study (36) and one of the key points in inhibiting early biofilm formation (37). We assumed that this initial burst was related to the diffusion of vancomycin located near the surface of the composites. Since the TCP particles were only physically blended into the polymer, they occupied random spaces in the polymer. After the composite was immersed in solution, the hydrophilic TCP particles tended to fall off and interact with the surrounding medium. The falling of TCP also created voids within the composite, thus exposing their surfaces to hydrolytic attack and weakening the overall structure.

Crystal violet is a dye that generally binds to biofilm polysaccharides and make biofilm visible (38). According to crystal violet staining results, vancomycin containing PDLLA/β-TCP and PLGA/β-TCP composites were able to inhibit early biofilm formation (39) and, therefore, preventing early biofilm formation was critical in the treatment of osteomyelitis (2). Since the protocol was done with planktonic bacteria, there was no statistically significant difference between the time points.

The proliferation of MSC and SaOS-2 cells with the composites was established, and this finding was in line with previous studies (40, 41) where cells were combined with other biomaterials. However, there was no correlation between the proliferation rate and the topography of the composite surfaces, since the smooth surface structure led to a lower cell proliferation rate with respect to the study conducted by Pulyala et al. (42).

Cells interacted with composites presented more ALP activity than the cells cultured without any composite, but there was no significant difference between the groups for hBM-MSCs. The significant differences in the SaOS-2 cell groups were related to the osteoblast-like nature of the SaOS-2 cells. Since these cells had osteoblast-like properties, it was expected that these cells showed a higher ALP activity than hBM-MSCs (43). Our findings were in line with previous studies (44, 45).

ALPL, however, decreased in the composite groups. The expression of ALPL was low for both composites, but the differences in the fold changes were not significant. The cells cultured with the osteogenic medium showed a higher ALPL expression with regard to the presence of the dexamethasone and ascorbic acid (46) found in the osteogenic medium. BMP1, a secreted metalloprotease requiring calcium and necessary for cartilage and bone formation (47), was significantly upregulated in the osteogenic medium group, opposed to the composite groups. The expression and activation of RUNX2 (48) is regulated by many bone-derived growth factors, including BMPs. BMPs form a unique group of proteins within the TGF-β super family of genes and play pivotal roles in the regulation of cartilage and bone development. BMP-activated SMADs (SMAD1, -5, and -8) induce *RUNX2* gene expression, and SMADs interact physically with the RUNX2 protein to induce osteoblast differentiation (49). In our study, neither BMP1 nor BMP4 was upregulated. Only BMP2 was upregulated with the composites, but this upregulation was not sufficient for inducing the upregulation of SMADs, and consequently, there was no RUNX2 upregulation (50). On the other hand, TGF-β1 upregulated in the composite groups, but this still did not lead to the upregulation of the *SMAD* genes. The upregulation of RUNX2 in the osteogenic medium group, however, had no statistically significant difference when compared with the composite groups. TGF-β2, one of TGF-β isoforms within the bone matrix, modulates the differentiation of osteoblasts and the proliferation of osteoprogenitor cells (51). Here, only the cells cultured with osteogenic differentiation medium showed the upregulation of TGF-β2, but this upregulation was not significant. On the other hand, it was downregulated in the composite groups and, therefore, osteoblastic differentiation of the cells in the composite groups could have been delayed.

Composite groups did not present any osteoinduction activity according to the qRT-PCR studies as they did not cause hBM-MSCs to express a group of osteogenesis-related signaling molecules in the absence of an osteogenic medium (48). In addition, the high content of TCP in the composites may inhibit the expression of some osteogenic markers by hBM-MSCs (52). The composites, thus, showed a higher ALP activity with the colorimetric assay as a sign of mineralization. This could be a feature of high TCP content in the composites (53).

In conclusion, we were able to produce and characterize biocompatible PDLLA/β-TCP and PLGA/β-TCP composites that were sufficiently released vancomycin *in vitro*. These composites inhibited early biofilm formation and allowed MSC and SaOS-2 cell proliferation. Osteogenesis was not achieved as these composites were osteoconductive. Combining these composites with osteogenic active molecules could be a strategy for future studies.

## Data Availability

The original contributions presented in the study are included in the article/Supplementary Material; further inquiries can be directed to the corresponding author/s.
